# A 12-month, moderate-intensity exercise training program improves fitness and quality of life in adults with asthma: a controlled trial

**DOI:** 10.1186/s12890-015-0053-8

**Published:** 2015-05-07

**Authors:** Andreas Meyer, Sabine Günther, Timm Volmer, Karin Taube, Hans J Baumann

**Affiliations:** Department of Pneumology, Kliniken Mariahilf GmbH, Mönchengladbach, Germany; Department of Pneumology, University Medical Centre Hamburg-Eppendorf, Hamburg, Germany; SMARTSTEP Consulting, Hamburg, Germany; Atem-Reha, Hamburg, Germany

**Keywords:** Asthma, Adult, Exercise, Physical fitness, Quality of life

## Abstract

**Background:**

Physical training has been shown to improve exercise capabilities in patients with asthma. Most studies focused on children and younger adults. Previously, the maximum program duration was six months. It is not known whether the same results may be obtained with lower intensity programs and sustained for time periods longer than 6 months. This controlled study was undertaken to investigate the effects of a moderate intensity outpatient training program of one year duration on physical fitness and quality of life in adults with asthma.

**Methods:**

21 adult asthmatics (mean age 56 ± 10 years) were allocated to outpatient training (n = 13) or standard care (n = 8). Exercise consisted of once weekly, 60-minute sessions of moderate intensity. Assessments at baseline and after one year included cardiopulmonary exercise testing and Short Form-36 and Asthma Quality of Life Questionnaires.

**Results:**

Following one year of exercise, relevant improvements were observed in the training group for maximum work capacity (p = 0.005), peak oxygen uptake (p < 0.005), O_2_pulse (p < 0.05), maximum ventilation (p < 0.005), and most of the quality of life domains. No changes were observed in the control group.

**Conclusions:**

A physiotherapist-led, long-term, moderate-intensity exercise program of one year duration can induce clinically relevant improvements in exercise capabilities and health-related quality of life in well-motivated adults with asthma.

**Trial registration:**

clinicaltrials.gov NCT01097473. Date trial registered: 31.03.2010.

## Background

Asthma is a chronic disease that is characterized by reversible bronchial obstruction leading to dyspnea and limited exercise capabilities [1]. Asthmatics often struggle to distinguish breathlessness associated with bronchoconstriction from breathlessness caused by exercise-induced hyperventilation. Patients may develop anxiety about both experiences leading to a sedentary lifestyle and subsequently lack of fitness. This mechanism is illustrated by the observation that exercise capabilities in asthmatics are related to habitual activity rather than airflow obstruction or bronchial hyperreactivity [2]. Adult patients with asthma are less fit than their peers [3].

Physical training in general is well known to support a healthy lifestyle. Regular aerobic exercise improves cardiovascular function, reduces mortality, and leads to a variety of psychological and sociological benefits [4]. As in healthy individuals, regular physical training improves health in people with asthma: Therefore, current guidelines incorporate the recommendation for all patients with asthma to engage in regular physical activity [1,5].

A recent Cochrane analysis showed positive effects of physical training on physical fitness in asthma whereas only limited data regarding health-related quality of life was found [6]. Only studies with at least twice weekly sessions were included. In most studies on exercise training in asthma the interventions took place in specialized rehabilitation clinics and lasted from 3 weeks up to no more than 6 months. The intensive use of resources involved may impede the transfer of the published results into real life in the light of limited economical resources.

While a significant proportion of the adult population suffers from asthma, the published studies included predominantly children or young adults [7,8]. A consensus report emphasized the need for research on the effects of pulmonary rehabilitation especially in elderly asthmatics [9]. As yet, no program has been evaluated which offers regular physical training for one year or more to adult asthmatics in an outpatient setting.

For this reason an inexpensive outpatient training program for adult patients with asthma was developed. Its goal was to encourage inactive individuals to start and continue physical training. The hypothesis was that this long-term training program of once-weekly sessions would improve cardiorespiratory fitness and quality of life in adults with asthma.

## Methods

### Participants

A prospective pseudo-randomized controlled study of adults with asthma was carried out. Patients were included if they had not participated in a pulmonary rehabilitation program in the past 12 months. Inclusion and exclusion criteria were adopted from the German Airway League recommendations [10]: Patients with postbronchodilator FEV_1_ < 60% predicted, history of recurrent exacerbations, hypoxemia (PaO_2_ < 50 mm Hg), hypercapnia (PaCO_2_ > 45 mm Hg), history of decompensated cor pulmonale, uncontrolled coronary heart disease, uncontrolled arterial hypertension, no history of hemodynamically relevant cardiac rhythm disorders, severe osteoporosis or other comorbities limiting exercise capabilities, severe overweight (body mass index > 35 kg/m^2^), were excluded. Patients unable or unwilling to attend training sessions regularly were also excluded. Patients were excluded, if they were unable tolerate a minimum work with the equivalent of 50 Watt on a cycle ergometer. This test was performed as needed by the referring pulmonary specialist prior to referral. There was no upper limit of exercise capabilities as an exclusion criterion.

A newspaper article was used to recruit subjects in the Hamburg metropolitan area (a city of 1.7 million inhabitants). Patients were allocated to the intervention or control group depending on where the patients lived: patients living within 7 km of the training locations formed the intervention group while those living further away served as controls.

All participants had a diagnosis of asthma according to standard criteria [11]. The patients’ pulmonary specialists confirmed the diagnosis and were responsible for their medical treatment, which had to be consistent with standard recommendations. The determination whether allergic asthma was present was made by the referring pulmonary specialists which included assessment of the medical history, serological and skin prick testing.

The local ethics committee of the Chamber of Physicians of Hamburg approved the study protocol. All patients agreed to participate in the study and gave their written informed consent.

### Training program

Study participants were allocated to three previously established outpatient pulmonary training groups run in cooperation with local sports clubs. Training was carried out in groups of 8–12. Weekly sessions ran for 60 minutes in the late afternoon. Sessions were held in an indoor gym equipped with simple training devices such as gymnastic balls, elastic bands and benches. All trainers were educated in exercise therapy and respiratory physiotherapy. One of two physicians (A.M., K.T.) was present during training sessions. Peak flow was measured before and after each session. Participants were asked to use their inhaled bronchodilators before each session prior to peak flow measurements. Each participant kept a training log to record attendance rates and peak flow measurements.

The training program was standardized across all groups. A 15-min. warm-up period of walking at different speeds accompanied by light exercises of different muscle groups was followed by endurance and circuit training including upper and lower extremities for 30 minutes. Participants were encouraged to exercise at a cardiac frequency > 60% of the maximum heart rate reached during the initial cardiopulmonary exercise test. Such training intensity is generally rated as moderate [12]. Patients monitored their own heart rate. Participants were advised to use diaphragmatic breathing and pursed lip breathing to improve ventilation. The last 15 minutes included a cool down phase with progressive stretching and muscle relaxation. No rhythm or music was used during the sessions to allow participants to exercise at their own pace. Subjects were encouraged to increase physical activity at home, i.e. to walk instead of drive, climb the stairs instead of using the elevator etc. Apart from the training sessions no other interventions such as education or counselling were included in the study program. The control group did not receive any intervention.

### Assessments

All participants were tested at baseline and after 12 months. Lung function including inspiratory vital capacity was obtained in the referring pulmonary specialists’ practices. All cardiopulmonary exercise testing was performed in one pulmonary specialist practice. The investigator (S.G.) was blinded to group status. An unsteady state cycle ergometer test (Oxycon alpha, Viasys, Würzburg, Germany) was performed with work increments of 10 watts each minute until exhaustion.

At the same time points, all participants were asked to complete two self-administered quality of life questionnaires. We used a German validated version of the short form 36 (SF-36), a questionnaire covering general aspects of health-related quality of life [13]. Scores range from 0 (worst possible health) to 100 (best possible health status). Clinically important differences were determined from published recommendations [14].

**Figure 1:**
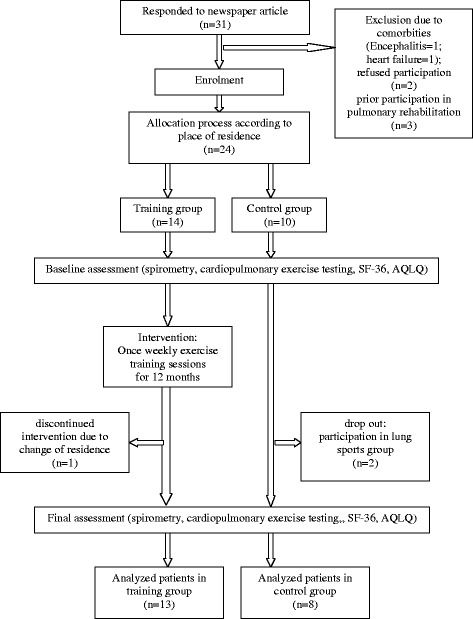
Study design (according to the CONSORT statement).

The second instrument was the validated German version of the disease specific Asthma Quality of Life Questionnaire (AQLQ) [15]. The response options for each of the 32 items in four domains are on a 7-point scale where 1 indicates maximal impairment and 7 indicates no impairment. A change > 0.5 points was rated as clinically significant as recommended [16]. See Figure 1 for patient flow.

### Sample size

The sample size was based on a power calculation using maximum oxygen uptake (V_O2max_) as the primary outcome measure (effect size, 1.16; standard deviation [SD], 4.0 L/min). V_O2max_ was chosen as the primary outcome measure because it is a valid and reliable measure of exercise capabilities. The estimate of the effect size was based on previous studies on exercise training in pulmonary rehabilitation [17]. The estimated number of patients required in each group to detect a difference in V_O2max_ with 80% power, a 2-tailed test, an α level of *P* less than .05 and an allocation ratio of 0.8 was 10 patients in the intervention group and 8 patients in the control group. To allow for a 10% dropout rate, additional patients were recruited.

### Statistical analysis

Data were analysed for statistical significance using SPSS 7.5. Results were reported as means with standard deviation (SD). Normality was tested using the Kolmogorov-Smirnov-test. A paired *t*-test was used to compare measurements made at the start (t1) and following one year of physical training (t2) in each group and a two-sample *t*-test for unequal variances. Effects were considered significant at p < 0.05.

## Results

### Patient population

Thirty-one patients responded to the newspaper article. Six subjects had to be excluded. Of the remaining 24 patients, 14 were assigned to the training program and 10 served as controls. One patient in the active group moved away and did not complete the study. Two patients in the control group wanted to participate in the active lung sports group and were consequently excluded from the study. Characteristics of the 21 participants (13 women, 8 men, mean age 57 ± 10 years) completing the study are shown in Table 1. All results refer to this population. There were no differences between the groups in age, duration of disease, lung function or medication. In the exercise group, 6 subjects had mild, 4 moderate, and 3 severe asthma. The control group included 2 subjects with mild, 3 with moderate, and 3 with severe asthma.

**Table 1:** **Baseline characteristics of the study population (n = 21)**

	**Training group**	**Control group**	**Group differences**
Number	13	8	n.s.
Sex (F/M)	8/5	5/3	n.s.
Age (years)	54 ± 11	59 ± 9	n.s.
Duration of disease (years)	19 ± 22	21 ± 15	n.s.
Allergic asthma	5/13	4/8	n.s.
Body mass index (kg/m^2^)	23 ± 1.6	22 ± 2.1	n.s.
VC (L)	3.4 ± 0.9	3.0 ± 0.8	n.s.
FVC (L)	3.3 ± 0.9	3.0 ± 1.0	n.s.
VC %pred	89 ± 14%	80 ± 14%	n.s.
FEV_1_ (L)	2.2 ± 0.8	2.0 ± 0.7	n.s.
FEV_1_ %pred	74 ± 24%	67 ± 16%	n.s.
FEV_1_ / FVC (%)	0.66 ± 0.092	0.66 ± 0.085	n.s.
Medication			n.s.
ICS (mg/d)	0.85 ± 0.4	0.8 ± 0.4	n.s.
long acting beta agonist	6/13	3/8	n.s.
montelukast	2/13	1/8	n.s.

Values are mean ± standard deviation.

F: female.

FEV_1_: forced expiratory volume in one second in litre/% of predicted.

ICS: inhaled corticosteroids (relative to beclomethasone).

M: male.

n.s.: not significant.

OC: intermittent use of oral corticosteroids during the study period.

VC: vital capacity.

The follow-up assessment regarding lung function and testing and body mass index showed no statistically differences compared to baseline assessments in both groups.

### Exercise tolerance

Cardiorespiratory performance at peak exercise for patients undergoing training and controls is shown in Table 2. All 21 participants had two cardiopulmonary exercise tests over a period of 12 month. At baseline (t1), reduced maximum work rates (WR_max_) and maximum oxygen uptake (V_O2max_) demonstrated the participants’ low level of fitness. WR_max_ was only 68% of predicted. Those assigned to the exercise group improved their mean WR_max_ by 18 ± 18 watts (p = 0.005), their mean V_O2max_ by 4.6 ± 4.36 ml/min/kg (p < 0.005), their oxygen pulse by 1.6 ± 1.98 ml (p < 0.05), and their minute ventilation (V_E_) by 13.2 ± 18.72 l/min (p < 0.005) after one year (t2). WR_max_ was different after one year between the two groups (p < 0.05). In the control group, no changes of V_O2max_, maximal V_E_, or oxygen pulse were observed after 12 months.

**Table 2:** **Peak exercise tolerance of training and control group before (t1) and after (t2) one year**

	**Training group**			**Control group**		
	**t1**	**t2**	**p**	**t1**	**t2**	**p**
	**(n = 13)**	**(n = 13)**	**t1 vs. t2**	**(n = 8)**	**(n = 8)**	**t1 vs. t2**
WR (Watt) ∑	97 ± 32	116 ± 31	0.005	107 ± 22	100 ± 28	0.480
VO_2_ (ml/kg/min) +	15.7 ± 5.0	20.4 ± 4.0	0.004	15.8 ± 4.0	16.8 ± 6.2	0.452
VO_2_/HR (ml)	8.7 ± 3.6	12.2 ± 3.9	0.032	9.4 ± 2.7	11.0 ± 2.5	0.108
V_E_ (l/min) +	38.2 ± 17.3	51.5 ± 20.3	0.002	42.1 ± 10.0	42.1 ± 10.3	0.993

Values are mean ± standard deviation.

No statistically significant differences between both groups at t1.

∑ p < 0.05 between both groups at t2.

n.s.: not significant.

V_O2_/HR: O_2_pulse.

V_E_: minute ventilation.

V_O2_: oxygen uptake per kg body weight.

WR: work rate.

+ p < 0.1 between both groups at t2.

### Health-related quality of life

Results of health status assessments are shown in Table 3. At baseline (t1) patients in both groups showed reduced quality of life according to their SF-36 and AQLQ scores. Complete quality of life data were obtained from all study participants at the final assessments (t2). Following the training program there were improvements in the SF-36 variables physical (+8.8 ± 10.7; p < 0.05) and social functioning (+11.5 ± 13.5; p < 0.05) for the training group (Table 3). Both changes were close to the published threshold for a clinically important difference. Regarding AQLQ, we found statistically and clinically significant improvements in the domains: activities (+0.8 ± 0.8; p < 0.05), emotions (+0.8 ± 1.2; p < 0.05) and overall quality of life (+0.6 ± 0.9; p < 0.05). The symptoms domain did not reach the level of statistical significance (+0.6 + 1.2; p = 0.093). All parameters remained unchanged in the control group after one year.

**Table 3:** **Health-related quality of life of training and control group before (t1) and after (t2) one year**

	**Training group**			**Control group**		
**SF-36**	**t1**	**t2**	**p**	**t1**	**t2**	**p (t1 vs t2)**
	**(n = 13)**	**(n = 13)**	**(t1 vs t2)**	**(n = 8)**	**(n = 8)**	
Physical functioning	62 ± 26	71 ± 20	0.016	69 ± 21	66 ± 28	0.752
Social functioning	67 ± 26	79 ± 22	0.014	67 ± 27	73 ± 22	0.703
Role limitations: Physical	23 ± 23	31 ± 18	0.256	14 ± 20	21 ± 22	0.200
Role limitations: Emotional	48 ± 44	58 ± 47	0.515	38 ± 38	62 ± 45	0.172
Mental health	55 ± 21	64 ± 13	0.119	56 ± 14	63 ± 23	0.270
Energy/vitality	46 ± 25	54 ± 18	0.418	41 ± 15	54 ± 24	0.140
Pain	70 ± 28	86 ± 17	0.129	79 ± 35	64 ± 29	0.230
General health perspective	47 ± 20	50 ± 16	0.650	44 ± 21	49 ± 28	0.288
**AQLQ**						
Overall QOL	4.3 ± 1.2	5.0 ± 1.1	0.027	4.3 ± 1.1	4.8 ± 1.5	0.270
Activities	4.0 ± 1.4	4.8 ± 1.3	0.009	3.9 ± 1.1	4.2 ± 1.7	0.691
Symptoms	4.2 ± 1.2	5.0 ± 1.1	0.093	4.4 ± 1.2	4.9 ± 1.5	0.222
Emotions	4.8 ± 1.8	5.6 ± 1.4	0.047	4.7 ± 1.3	5.3 ± 1.6	0.1
Environment	4.6 ± 1.4	5.1 ± 1.4	0.187	4.6 ± 1.6	5.2 ± 1.7	0.416

Values are mean ± standard deviation.

No statistically significant differences between both groups at t1.

AQLQ: Asthma Quality of Life Questionnaire, n.s.: not significant.

QOL: Quality of life, SF-36: Short form-36 questionnaire.

There was no relationship between improvements in exercise capabilities and the improvements in SF-36 or AQLQ scores at the end of the study.

### Peak flow measurements and adverse events

Patients used their medication regularly and peak flow measurements were constant over one year. A slight increase in peak flow rates was observed after each training session. No adverse events were noted during training sessions. Inhaled corticosteroid dose and bronchodilator use was unchanged to the baseline status in both groups. During the study period 2 patients in the training group and 4 patients in the control group needed the transient administration of oral corticosteroids.

### Adherence to training sessions

Adherence was 75 ± 15% during the study period. Most common reasons were personal (other engagement, holiday, social events) and to a much lesser degree exacerbations. The only patient who dropped out of the active group moved away.

## Discussion

The present study showed that adults with asthma participating in a long-term, outpatient training program experienced improved cardiorespiratory fitness and health-related quality of life. These results were achieved by a program of comparatively low intensity. A unique feature of the study in comparison to previous studies of physical training in asthma is its duration of one year.

Optimal asthma care should lead to a normal life without restrictions [18]. This includes adequate medication, patient education on disease self-management, and psychological support. In addition, regular physical activity provides benefits in asthmatic patients: Beneficial effects have been reported in a number of studies on children and young adults using in- and outpatient training programs.

The present study differs from those previously published in three important aspects. First, the training program duration was the longest ever reported for a controlled trial of exercise training in patients with asthma. Previous reports used programs of 6 to 16 weeks duration [7,19,20]. It is well known that effects gained by pulmonary rehabilitation programs wane subsequently [21]. Rather than achieving greater improvements of limited duration, training programs in chronic diseases should aim for lasting effects. The optimal duration of exercise programs is currently unknown. However, current guidelines recommend the longest duration possible and practical [21]. Whether it is possible in training programs using three sessions per week to keep adherence of patients with asthma above the suggested level of 75% for more than 16 weeks has not been shown before. Hence, extrapolation of the results found in previous, shorter trials might be problematic. The latest Cochrane review on physical training in asthma addresses this aspect and explicitly stresses the specific need for studies of longer duration, i.e. one year or more [6].

However, mechanisms explaining improvements of short to medium-term programs (i.e. up to 16 weeks) may not be the same as those responsible for the changes reported in the present study: In the long-term setting adherence and psychological effects may become more important than training intensity or frequency. In this respect, a study that followed young asthmatics after attending a 10-week rehabilitation program is noteworthy [22]. Patients were encouraged to undertake physical training at home but training intensity was left up to them. Interestingly, half of the patients decided to exercise 1–2 times per week but this was sufficient to maintain the initially achieved improvements over three years. This result highlights the assumption that over a longer period of time training once a week appears appropriate and feasible for most patients.

Second, the present study investigated effects of physical training in adults with asthma with a mean age of 56 ± 10 years. Apart from very few previous reports [19,20,23] only children or young adults have been investigated so far [7,8]. As asthma is a common diagnosis in adults this lack of data is surprising but may be due to underdiagnosis of adult asthma [9]. The only reports that studied older asthma patients differed from the present study as they either used breathing exercises as only training element [23], used a design of considerably higher intensity than in the present study [20] or failed to induce improvements in exercise capabilities [19]. Exclusion criteria especially regarding certain comorbidities may represent a possible reason for the paucity of data on exercise training in elderly asthma patients. The present study confirms that exercise training is feasible and effective not only in young but also in older patients in asthma.

Together with the studies from Turner et al. [19] and Boyd et al. [20] the present report is the first controlled trial that measured quality of life in a group of adult asthmatics participating in physical training. All study participants had a reduced quality of life according to SF-36 and AQLQ at baseline comparable to reported results for adults with asthma [24]. After one year of exercise training, components of SF-36 and AQLQ increased to a clinically meaningful extent [16,25]. In contrast to this study, Turner et al. only found improvements in quality of life while exercise capacity remained unchanged [19]. Boyd et al. demonstrated increments both in quality of life and fitness levels, however their study used a higher training intensity and covered a shorter time period [20].

Previous controlled studies reporting quality of life data only studied children [26,27], young adults [7,8] or mixed groups of patients with asthma and COPD [28]. All reported improvements in some of the measured components. As in the present study, changes in exercise performance were not correlated with the corresponding changes in quality of life in all but one report [7]. This observation indicates that factors other than physical capabilities influence quality of life. Furthermore, physical training can also improve emotional scores. This fact underlines the important link between physical and emotional handicap in chronic diseases such as asthma and the psychological barriers against physical activity [2].

Third, in the present study a training program of lower intensity than usually recommended achieved the improvements. It is generally assumed that aerobic endurance training on fewer than 2 days per week, at less than 40-50% of V_O2max_, and for less than 10 min is not a sufficient stimulus for developing and maintaining fitness [29]. In contrast, the presented training group only had once weekly supervised sessions. Several circumstances may explain why this program still demonstrated increases in exercise capabilities similar to reported results [7,19,20].

The subjects started the program with a markedly reduced fitness reflected in a WR_max_ of 68% of predicted values, which is comparable to the range reported by Mendes et al. [7]. It has been shown that in COPD patients training of lower intensity and longer duration may be beneficial [30]. Furthermore, the cited guidelines were written for healthy individuals with the aim of increasing fitness to a high level in a reasonable time. Recognizing that these recommendations cannot always be followed by older people, the American College of Sports Medicine subsequently published a position statement on physical activity in older adults which recommends a lower intensity and frequency of exercise [31]. Disabled subjects with chronic diseases and reduced physical fitness might not wish to achieve the highest levels of fitness. For these patients it appears to be sufficient to get fit enough to permit daily living.

Exercise limitation in asthmatics is not correlated to disease severity but is largely due to psychological factors [2,32]. Patients may reduce their daily physical activity due to perceived dyspnea leading to a lower level of fitness. Once patients are used to certain levels of dyspnea, they may tolerate higher exercise levels independent of underlying lung function. It is well documented that physical training does not induce improvements in lung function [6]. In line with this observation an increase in maximum V_E_ without changes in lung function was found. In this study, patients trained under permanent supervision of a physician. Hence, patients felt more confident and exercised near their physical limits. Once they experience benefits from physical training, this may translate into a higher degree of activity in daily life.

Experiments in healthy twins have shown that intense training with several sessions per week can increase fitness rapidly with the disadvantage that this fitness is lost in the same time period [33]. Low intensity training once a week is a sufficient training stimulus and results in a relevant but much slower increase in maximal workload. “Athletic performance” which describes stability of fitness over time in this mode of training appears to be higher. This aspect is important for patients with chronic diseases who cannot attend training sessions regularly. In this study, training attendance was 75 ± 15% over one year.

Compared to published studies, the cost of establishing our training program was low. Using facilities at local sports clubs, it was possible to run our program at a cost of €10 per training session per participant. This low cost design improves the practicability of the presented program in the current health care environment with its financial restrictions. The ratio of 8–12 participants per single trainer was higher than usual. While it was impossible to offer each subject individualised training, this was balanced by higher motivation in a group of patients with common interests. Furthermore, every unit of the program allowed training on an individual level.

### Study limitations

This study has some limitations including the non-randomized allocation process. Intervention allocation was based on the patient’s place of residence for practical reasons. As there were only two training sites available only patients living nearby were included in the training group. It was felt that travelling long distances would have impeded long-term adherence since travel time is a well-known factor of non-adherence. However, the conclusions drawn are unlikely to be limited to a great extent by this methodological aspect. The allocation process resulted in equally distributed baseline characteristics in both groups. As all participants lived in the same urban area the social environment was comparable for all participants.

The high motivation in the training group was demonstrated by a high attendance rate and the fact that the only patient who dropped out had moved away. As motivation represents an important factor, one has to be careful when generalizing the results from a single group.

As the use of oral corticosteroids during the study period was higher in the control group than in the training group, the intergroup difference in exercise capabilities may be explained by a difference in exacerbation rates. Although it cannot be ruled out that this difference was purely by chance, we hypothesize that the lower exacerbation rate in the intervention group may reflect antiinflammatory effects of aerobic training. This hypothesis is supported by consistent reports of reduced airway inflammation following exercise training [34]. Furthermore, a prospective study found that in older women regular physical activity was associated with a reduced risk of asthma exacerbations [35].

We did not measure physical activity outside training sessions; total exercise time per week is unknown. As a consequence, we cannot determine whether the once weekly training sessions or general lifestyle changes led to the observed improvements. Future studies should therefore measure physical activity levels, both pre- and post-intervention.

It is important to notify that the positive effects on physical fitness achieved in our comparably small sample may not be generalized to the broader asthmatic population as it is well known that training effects are greater in less fit individuals. This correlation has been shown in the work of Mendes et al. who found an inverse correlation between baseline VO_2max_ and the training-related improvements in VO_2max_ [7].

## Conclusions

This study demonstrates that a long-term, moderate-intensity physical training program was associated with relevant improvements of fitness and health-related quality of life in well-motivated older adults with asthma. These effects were achieved in outpatient sport groups using limited resources. The duration of the program, one year, is the longest published so far.

## Abbreviations

AQLQ: Asthma quality of life questionnaire
SD: Standard deviation
SF-36: Short form 36 quality of life-questionnaire
t1: Baseline assessment
t2: Follow-up assessment after 12 months
V_E_: Minute ventilation
VO_2max_: Maximum oxygen uptake
WR_max_: Maximum work rate
